# Tabersonine ameliorates osteoblast apoptosis in rats with dexamethasone-induced osteoporosis by regulating the Nrf2/ROS/Bax signalling pathway

**DOI:** 10.1186/s13568-020-01098-0

**Published:** 2020-09-11

**Authors:** Xi Sun, Lijun Gan, Nan Li, Shuyi Sun, Na Li

**Affiliations:** 1 Department of Neonatology, First Hospital of Jilin University, No. 71 Xinmin Street, Chaoyang District, Changchun, 130021 Jilin China; 2 Department of Ultrasonography, Jilin Provincial People’s Hospital, Changchun, 130000 Jilin China

**Keywords:** Tabersonine, Dexamethasone, Osteoporosis, Osteoblast, Inflammation, Oxidative stress

## Abstract

We explored how tabersonine (Tab) protected against dexamethasone (Dex)-induced osteoporosis. Osteoblasts were treated with Dex (100 µM) with or without Table (5 or 10 µM). We measured cell viability, alkaline phosphatase (ALP) activity, and mitochondrial superoxide and reactive oxygen species levels. We used flow cytometry to explore the effects of Tab on mitochondrial membrane potential and osteoblast apoptosis. We used RT-PCR and western blotting to examine the effect of Tab on protein expression. We evaluated the effects of Tab on bone histopathology and bone mineral density in rats with Dex-induced osteoporosis. Tab increased cell viability and ALP activity, and reduced the mitochondrial superoxide, reactive oxygen species and matrix metalloproteinase levels and osteoblast apoptosis. Tab significantly reduced the levels of nuclear factor erythroid 2-related factor 2 (Nrf2), haem oxygenase-1 and NAD(P)H quinone dehydrogenase 1. Moreover, it increased the levels of mRNAs encoding runt-related transcription factor 2, bone morphogenetic protein-2 and osterix. These data suggest that Tab ameliorates Dex-induced osteoporosis by regulating the Nrf2 signalling pathway.

## Introduction

Osteoporosis is a metabolic bone disease characterised by the loss of bone mass and bone weakness; it affects about 200 million individuals (80% of whom are female) worldwide (Sözen et al. 2017). Skeletal integrity is compromised in osteoporotic patients; an imbalance develops between bone formation and resorption, triggering fracture (Office of the Surgeon General (USA) 2004). Osteoblasts are responsible for bone formation and mineralisation. Several factors regulate osteoblast activity. Oestrogen enhances osteoblastic mineralisation and stimulates osteoblast differentiation and proliferation (Domazetovic et al. 2017). In females, osteoporosis commonly develops after menopause or ovariectomy (Luo et al. 2014). Nuclear factor erythroid 2-related factor 2 (Nrf2) expression contributes to osteoporosis development by enhancing reactive oxygen species (ROS) production (Ma et al. 2013). Mitochondrial dysfunction enhances osteocyte apoptosis, reducing bone mineralisation via ROS overproduction (Smietana et al. 2010). The antioxidant responsive element, in combination with the Nrf2 activator, prevents osteoporosis development by enhancing the expression levels of antioxidant enzymes (Jin et al. 2020). Osteoblast activity is regulated by the Nrf2 pathway (Sun et al. 2015). Conventional osteoporosis treatments have several limitations; alternative medicines have shown promise over the past few decades.

Tabersonine (Tab), originally isolated from *Catharanthus roseus* (*Apocynaceae*), is a terpene indole alkaloid found in several medicinal plants (Almagro et al. 2015). *C. roseus* is traditionally used in China for the management of cancer, malaria and diabetes (Nejat et al. 2015). Tab has strong anticancer and neuroprotective effects (Nejat et al. 2015). It protects against lung injury by regulating the NF-κB and p38/MK2 pathways, thereby strongly inhibiting inflammation (Zhang et al. 2018). In this study, we explored whether Tab protected against dexamethasone (Dex)-induced osteoporosis, and the molecular mechanism in play.

## Materials and methods

### Animals

Neonatal Sprague-Dawley rats (strain P2) were sacrificed via decapitation and their calvaria were isolated for in vitro analysis. The in vivo study was performed with 8-week-old female rats (mean body weight 250 ± 25 g), held under standard laboratory conditions. All animal procedures were approved by the animal ethics committee of the First Hospital of Jilin University, China (Approval no. IAEC/FH-JU/2018/01).

### Chemicals

Tab was supplied by the Institute of Botany, Chinese Academy of Sciences, China; Dex was obtained from Tianjin Lisheng Pharmaceutical Co. Ltd., China. Alkaline phosphatase (ALP), Nrf2, and matrix metalloproteinase detection kits were purchased from Abcam, USA. The antibodies used for western blotting and the qRT-PCR primers were obtained from Thermo Fisher Scientific, USA.

### Osteoblast isolation and culture

Calvarial osteoblasts were isolated from neonatal rats as described previously (Liu et al. 2019). The connective tissue was cleaned carefully and digested with 0.3% (w/v) type II collagenase after isolation of the parietal bones. The cells were cultured in DMEM supplemented with antibiotics and antimycotics, charcoal dextran-treated foetal blood serum and non-essential amino acids (100 µM) for 48 h.

### Estimation of cell viability

Cell proliferation was estimated using the CCK-8 assay as described previously (Jo et al. 2015). Cells (2 × 10^4^/well) were seeded into six-well plates, incubated for 24 h, treated with 100 µM Dex with or without the addition of Table (5 or 10 µM) 2 h previously, and cultured for 24 h; CCK-8 was then added, followed by incubation for 2 h. Cell viability was determined by estimating absorbance at 450 nm using a microplate reader.

### Measurement of ALP activity

Osteoblasts (2 × 10^5^ cells/well) were seeded into six-well plates and treated with Dex with or without Tab for various durations. The cells were lysed with lysis buffer, and ALP levels were measured as directed by the kit manufacturer.

### Mitochondrial superoxide and ROS assays

Flow cytometry was used to measure mitochondrial superoxide and ROS levels in osteoblasts, with the use of the MitoSOXTM Red indicator and a nonfluorescent 2,7-dichlorofluorescin diacetate probe, respectively.

### Nrf2 assay

The TransAM Nrf2 assay was used to estimate Nrf2 activity, according to the manufacturer’s instructions. An ELX800 microplate reader was employed to measure absorbance at 405 nm.

### Mitochondrial membrane potential

Mitochondrial membrane potential (MMP) was estimated using the lipophilic, cationic fluorescent dye 5,5′,6,6′-tetrachloro-1,1′,3,3′-tetraethylbenzimidazol-carbocyanine iodide. Osteoblasts (2 × 10^5^ cells/well) were seeded into six-well plates and the Mitochondrial Membrane Potential Detection Kit was used to estimate MMP according to the manufacturer’s instructions.

### Osteoblast apoptosis

Osteoblasts (2 × 10^5^ cells/well) were seeded into six-well plates and treated with Dex with or without Tab for various durations. The cells were resuspended in 500 µl binding buffer containing PI (5 µl) and Annexin V-APC (5 µl), rinsed with PBS, and placed on ice. Flow cytometry was used to measure apoptosis.

### qRT-PCR

Osteoblasts were cultured with Tab for 48 h. Total RNA was extracted using the TRIZOL reagent and cDNA was synthesised using SuperScript II reverse transcriptase. qRT-PCR was performed using the SYBR Green PCR Master Mix system. GAPDH served as the internal control. Cycle number (the Cq value) was plotted against the log of the added cDNA concentration, and relative target gene expression levels were determined using the 2^−ΔΔCq^ method.

**Table:** 

BMP-2	Forward	5′-TGCGGTCTCCTAAAGGTCG-3′
	Reverse	5′-ACTCAAACTCGCTGAGGACG-3′
Runx2	Forward	5′-TCTCCAGGAGGACAGCAAGG-3′
	Reverse	5′-TTGCAGCCTTAAATGACTCGGT-3′
Osterix	Forward	5′-CACTCTCCCTGCCAGACCTC-3′
	Reverse	5′-GCCATAGTGAACTTCCTCCTCAAG-3′
β-actin	Forward	5′-GGAGATTACTGCCCTGGCTCCTA-3′
	Reverse	5′-GACTCATCGTACTCCTGCTTGCTG-3′

### Western blotting

Total osteoblast proteins were extracted into ice-cold radioimmunoprecipitation lysis buffer, and the DC Protein Assay was performed to estimate protein levels. Sodium dodecyl sulphate-polyacrylamide gel (10% w/v) electrophoresis was used to separate the proteins, which were then transferred to polyvinylidene difluoride membranes. The membranes were incubated with a 5% (w/v) fresh nonfat dry milk solution to block non-specific reactions. They were incubated at 4 ºC overnight with primary antibodies against Nrf2 (1:500), Ho-1 (1:1000), NAD(P)H quinone dehydrogenase 1 (NQO-1; 1:1000), caspase-3 (1:500), Bcl-2 (1:1000), Bax (1:1000), cytochrome-*C* (1:200) and β-actin (1:1000), and then at room temperature for 60 min with appropriate secondary antibodies. The enhanced chemiluminescence assay was used to detect protein bands, and the ImageLab software was employed for densitometric analysis.

### Osteoporosis induction

Rats were separated into four groups: control, Dex (1 mg/kg intramuscularly, daily for 60 days), and Tables 20 and 40 (20 and 40 mg/kg intraperitoneally, 30 min after Dex administration for 60 days). The animals were then sacrificed, and the bilateral femora were collected.

### Determination of bone mineral density

Dual energy X-ray absorptiometry was used to determine the bone mineral density (BMD). Rats were anesthetised with Zoletil/Rompun, and the femora, lumbar vertebrae and whole body were scanned for the evaluation of BMD.

### Histopathological analysis

The isolated tibiae were fixed in 10% (v/v) formalin at 40 °C for 2 days. Ethylenediaminetetraacetic acid (10% v/v) was used to decalcify the bones, which were then placed in liquid paraffin to create wax blocks. Sections (4 µm thickness) were prepared and stained with haematoxylin and eosin. Histopathological changes were identified under a tri-ocular microscope at 100× magnification.

### Statistical analysis

All data are expressed as means ± standard errors of the means (*n* = 10/group). One-way analysis of variance, followed by the Dunnett test, was performed using the GraphPad Prism software (ver. 6.1; GraphPad Software Inc., USA). *p* values < 0.05 were considered to reflect statistical significance.

## Results

### Effect of Tab on osteoclast viability

**Fig. 1:**
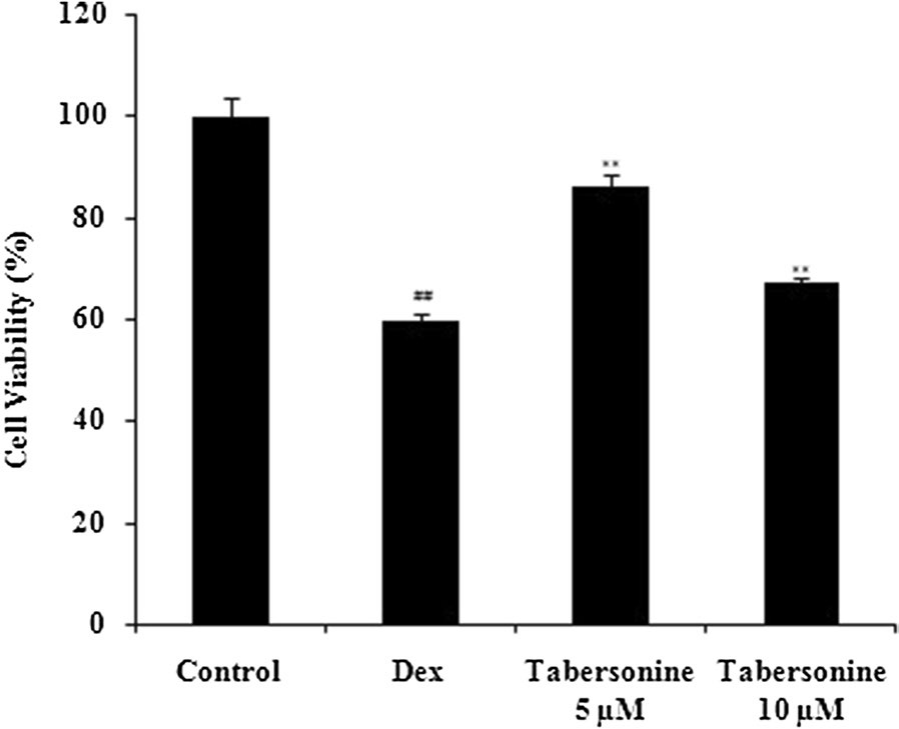
Effect of tabersonine on osteoblast viability after dexamethasone treatment, evaluated using the CCK-8 assay. Means ± SEMs (*n* = 10). ^##^*p* < 0.01 vs. control, **p < 0.01 vs. Dex group

Dex reduced osteoblast viability (compared with the control) and Tab rescued viability, as revealed by the CCK-8 assay (Fig. 1).

### Effect of Tab on ALP activity

**Fig. 2:**
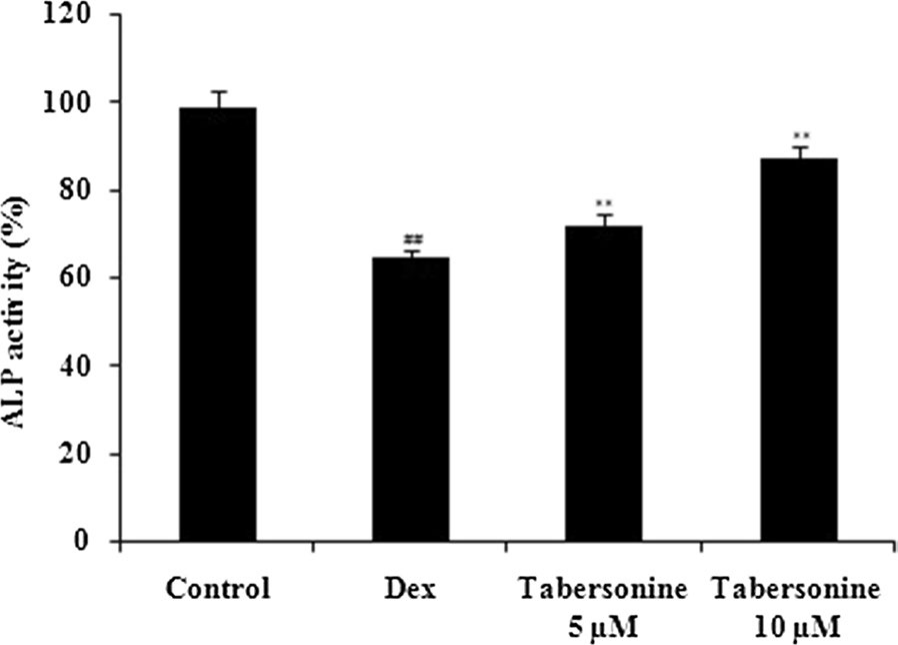
Effect of tabersonine on osteoblast alkaline phosphatase activity. Means ± SEMs (*n* = 10). ^##^*p* < 0.01 vs. control, **p < 0.01 vs. Dex group

Dex significantly reduced the osteoblast ALP level compared with the control (*p* < 0.01; Fig. 2), and Tab neutralised this negative effect of Dex.

### Effects of Tab on mitochondrial superoxide and ROS levels

**Fig. 3:**
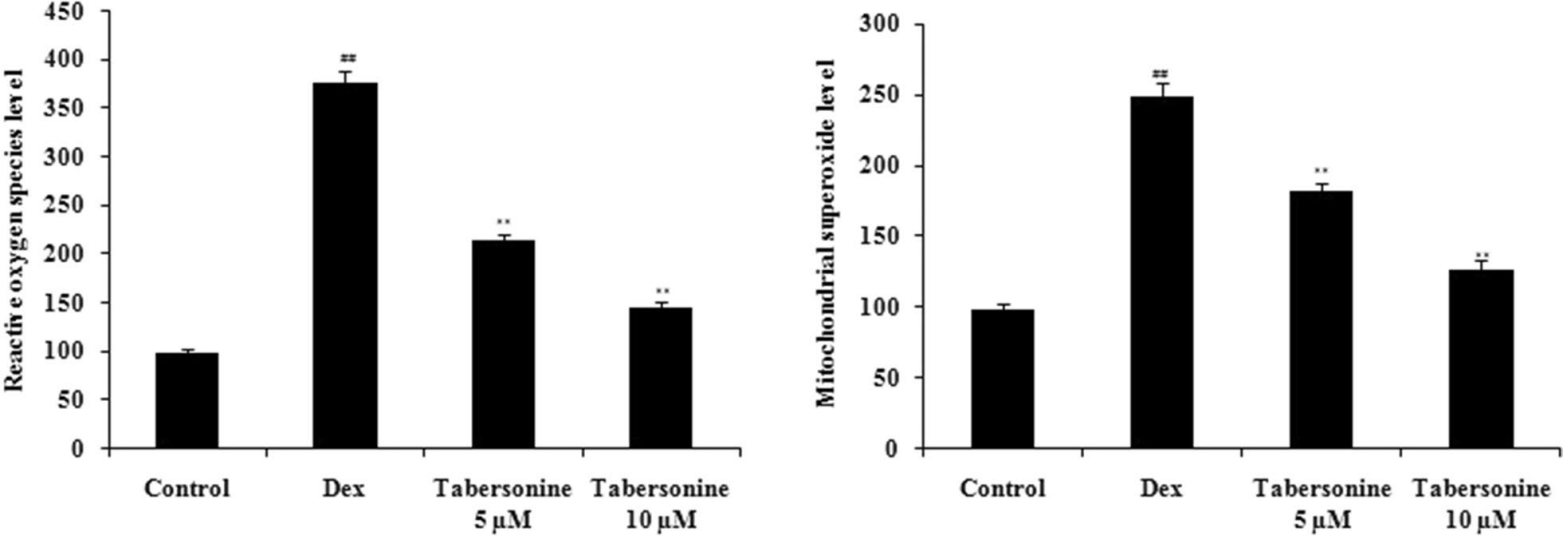
Effect of tabersonine on mitochondrial superoxide and reactive oxygen species production in osteoblasts, measured via flow cytometry. Means ± SEMs (*n* = 10). ^##^*p* < 0.01 vs. control, **p < 0.01 vs. Dex group

The effects of Tab on mitochondrial superoxide and ROS levels, as determined by flow cytometry, are shown in Fig. 3. Dex enhanced the production of mitochondrial superoxide and ROS compared with the control. Tab neutralised these negative effects of Dex.

### Effect of Tab on the MMP

**Fig. 4:**
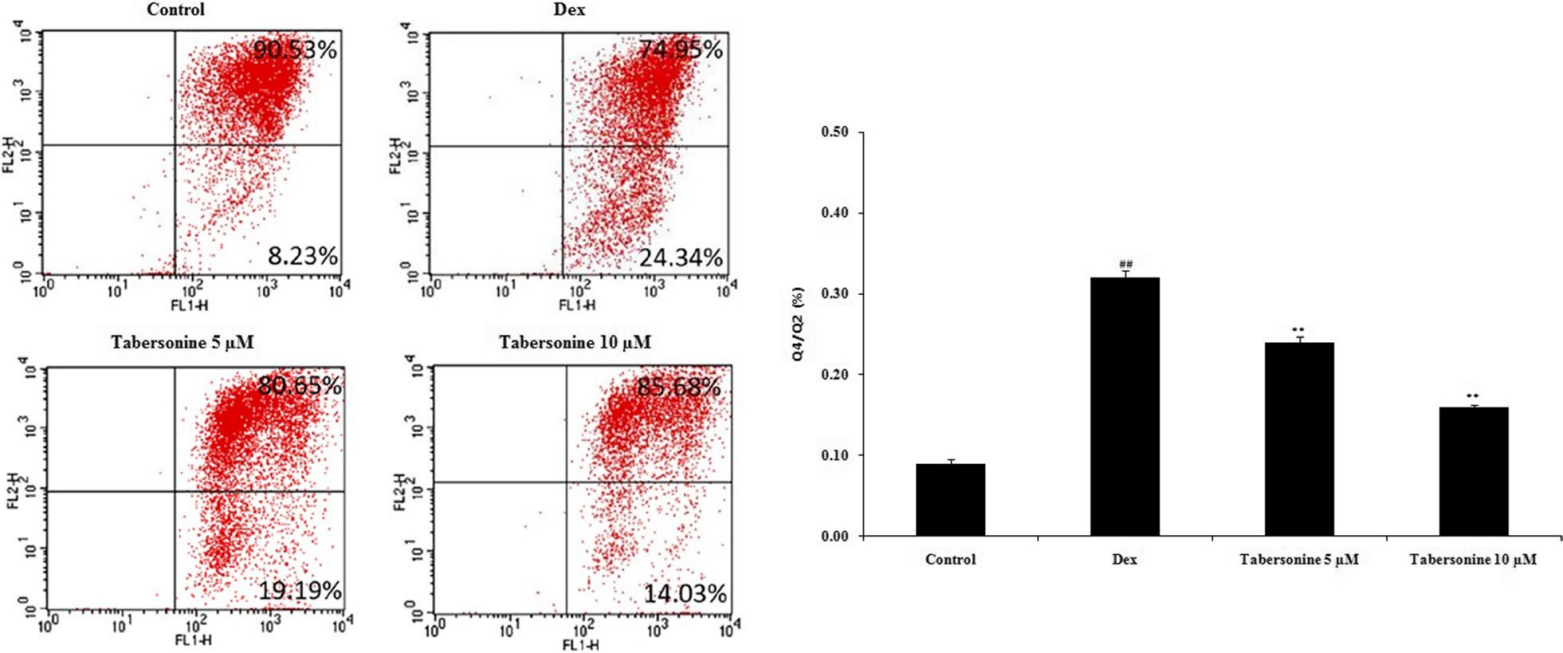
Effect of tabersonine on the mitochondrial membrane potential of osteoblasts treated with dexamethasone. Means ± SEMs (*n* = 10). ^##^*p* < 0.01 vs. control, **p < 0.01 vs. Dex group

The MMP was measured by estimating mitochondrial depolarisation via the change in the red/green fluorescence ratio (Fig. 4). The MMP was lesser in Dex-treated osteoblasts than in controls, and Tab neutralised this negative effect.

### Effect of Tab on osteoblast apoptosis

**Fig. 5:**
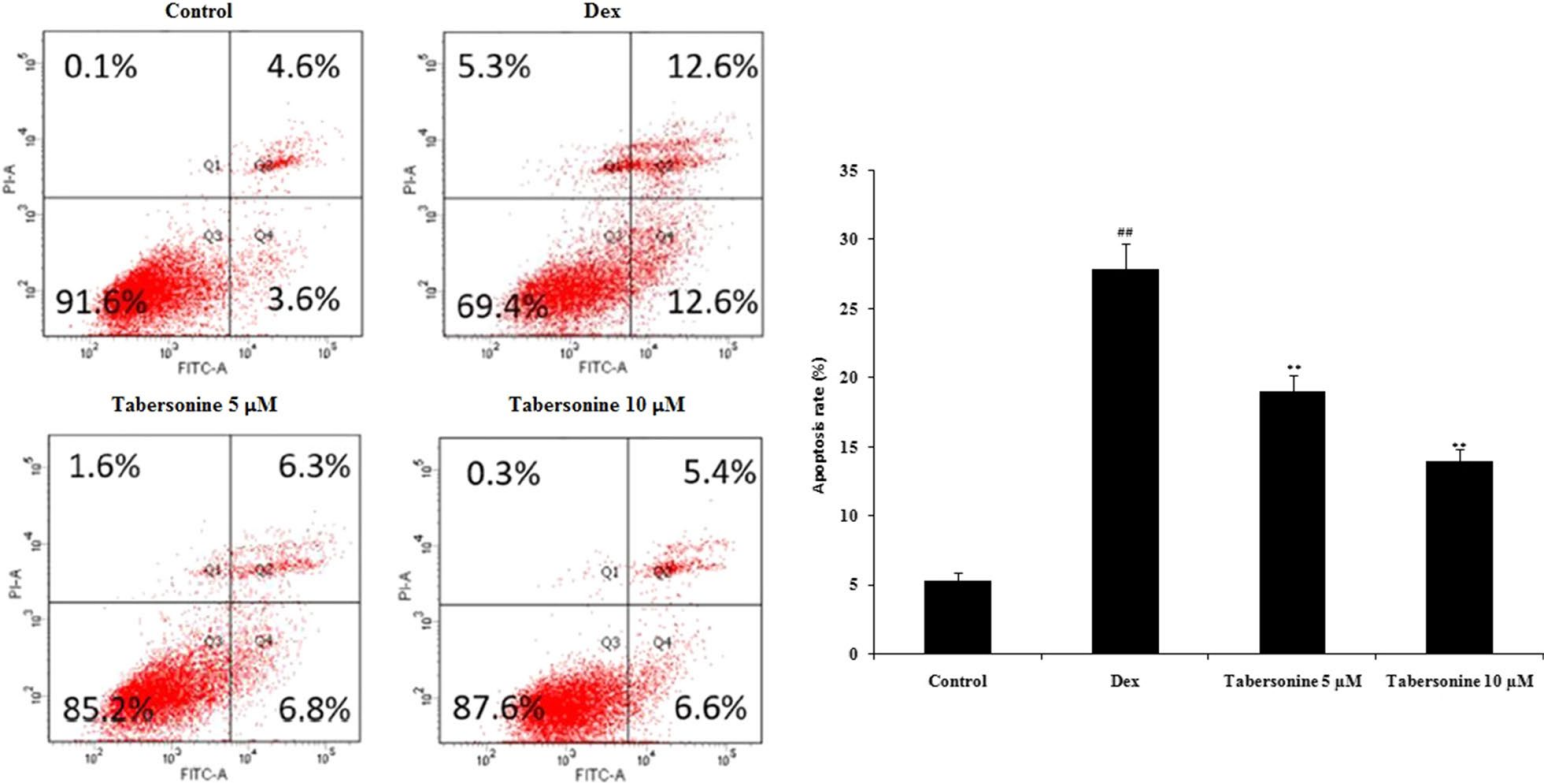
Effect of tabersonine on apoptosis of dexamethasone-treated osteoblasts. Means ± SEMs (*n* = 10). ^##^*p* < 0.01 vs. control, **p < 0.01 vs. Dex group

Osteoblast apoptosis was measured via flow cytometry (Fig. 5). Apoptosis was greater in Dex-treated than in control osteoblasts, and Tab neutralised this negative effect of Dex.

### Effects of Tab on the levels of mRNAs encoding runt-related transcription factor 2, bone morphogenetic protein-2 and osterix

**Fig. 6:**
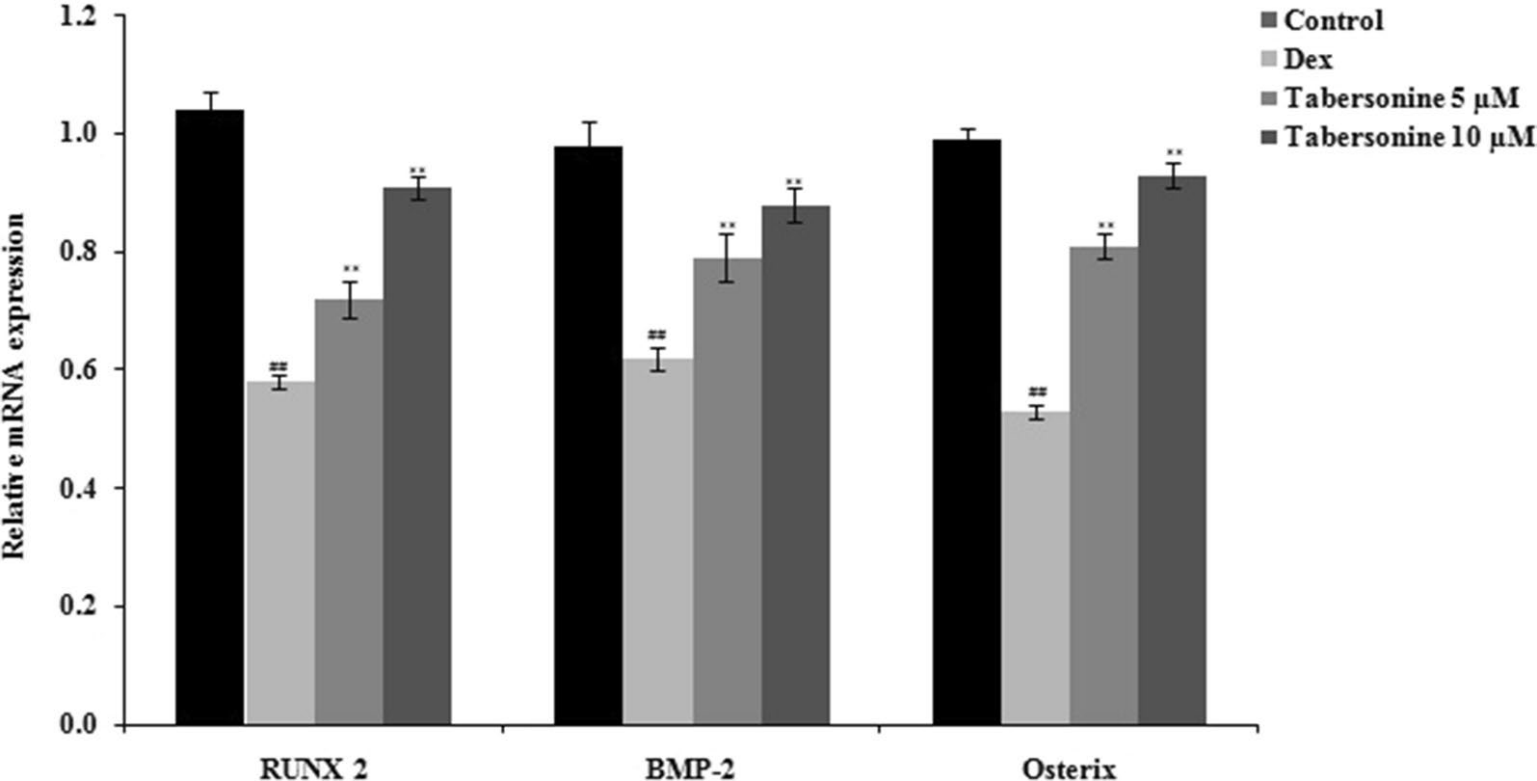
Effects of tabersonine on the levels of mRNAs encoding RUNX2, BMP-2 and osterix in dexamethasone-treated osteoblasts. Means ± SEMs (*n* = 10). ^##^*p* < 0.01 vs. control, **p < 0.01 vs. Dex group

The levels of mRNAs encoding runt-related transcription factor 2 (RUNX2), bone morphogenetic protein-2 (BMP-2) and osterix were measured in osteoblasts treated with Dex alone or Dex and Tab (Fig. 6). The mRNA levels decreased upon Dex treatment, and Tab neutralised these negative Dex effects.

### Effect of Tab on Nrf2 signalling

**Fig. 7:**
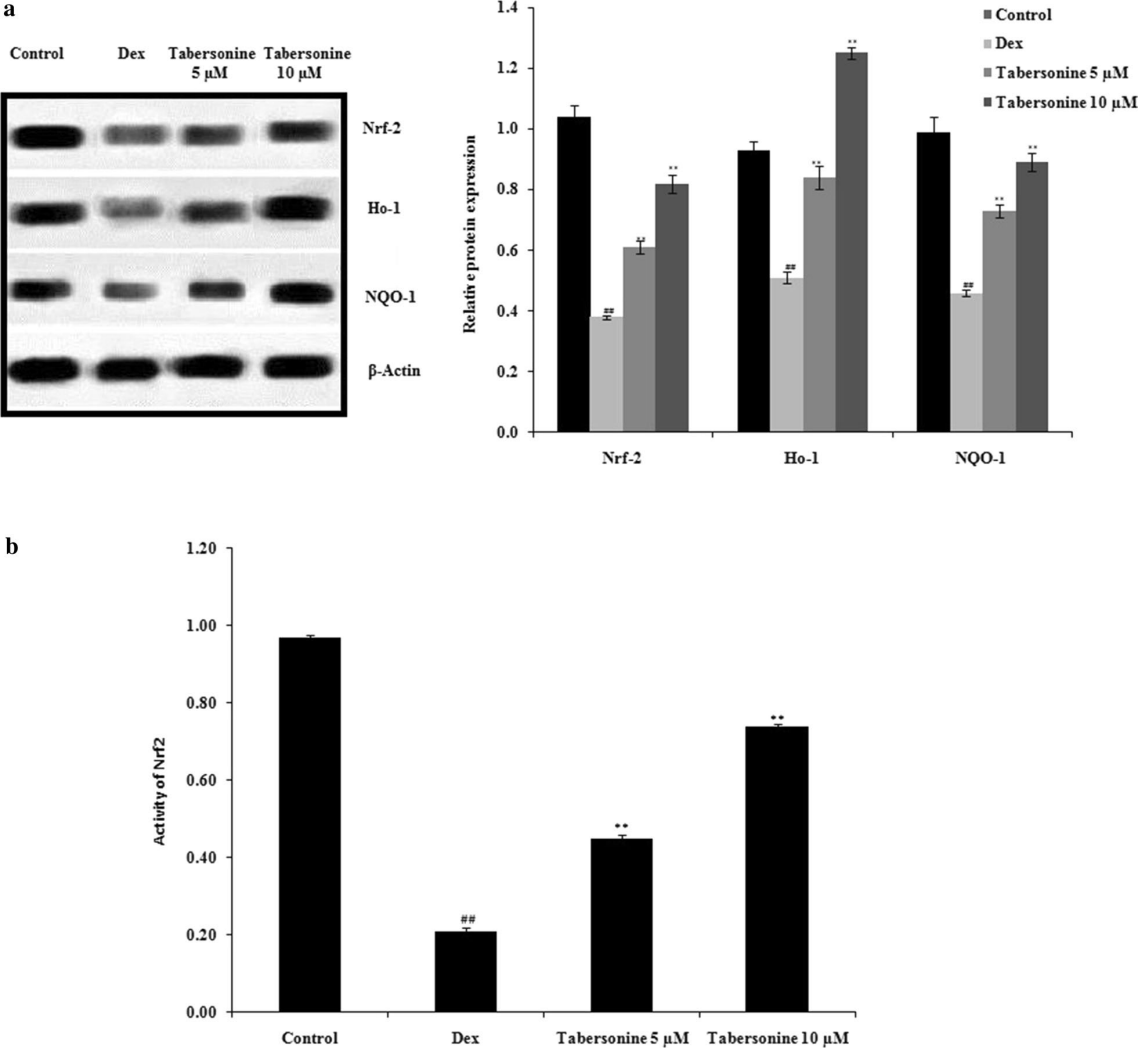
Effect of tabersonine on Nrf2 pathway activity in dexamethasone-treated osteoblasts. **a** Nrf2, Ho-1 and NQO-1 protein levels in osteoblasts, as revealed by western blotting. **b** Nrf2 activity, as determined using the TransAM Nrf2 Kit. Means ± SEMs (*n* = 10). ^##^*p* < 0.01 vs. control, **p < 0.01 vs. Dex group

The effect of Tab on Nrf2 signalling was evaluated by measuring the levels of Nrf2, Ho-1 and NQO-1 in osteoblasts via western blotting; Nrf2 activity was also determined using the TransAM Nrf2 kit (Fig. 7a, b). The Nrf2, Ho-1 and NQO-1 levels were lower in Dex-treated than in control osteoblasts. Tab significantly neutralised the negative effects of Dex (Fig. 7a). Nrf2 activity was reduced in Dex-treated cells compared with control cells. Again, Tab neutralised this negative effect of Dex (Fig. 7b).

### Effects of Tab on the expression of caspase-3, Bcl-2, Bax and cytochrome-*C*

**Fig. 8:**
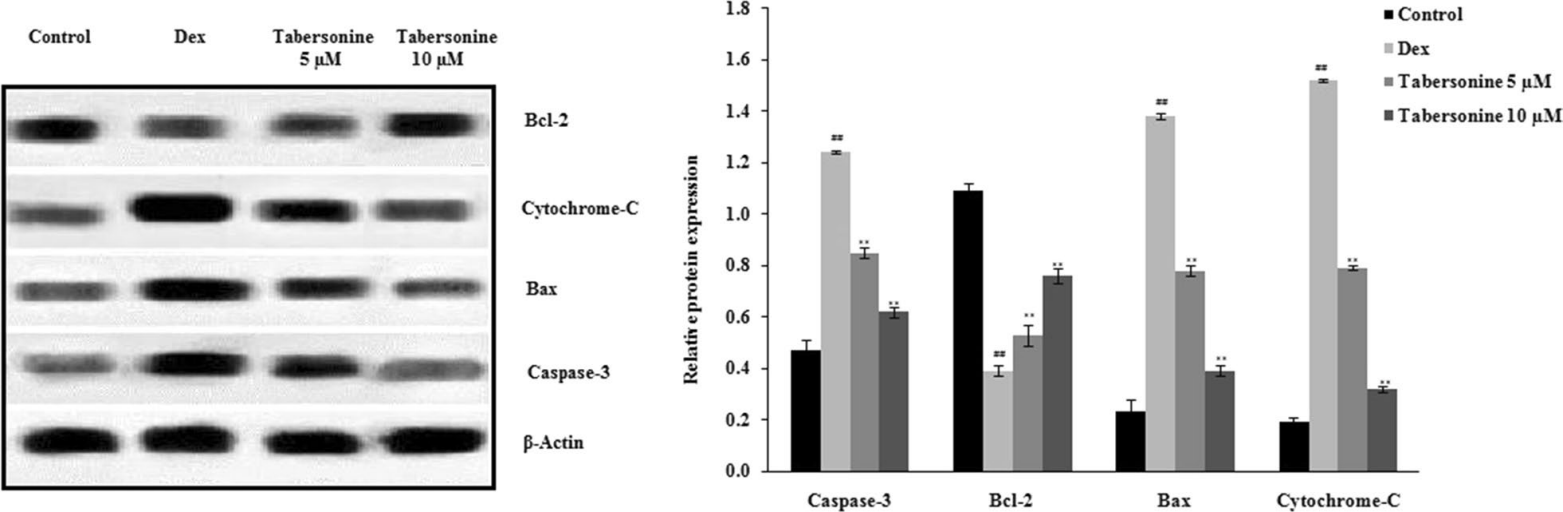
Effects of tabersonine on the levels of caspase-3, Bcl-2, Bax and cytochrome-*C* in osteoblasts. Means ± SEMs (*n* = 10). ^##^*p* < 0.01 vs. control, **p < 0.01 vs. Dex group

Tab affected the levels of pre- and pro-apoptotic proteins in Dex-treated osteoblasts (Fig. 8). Dex significantly increased the levels of caspase-3, Bax and cytochrome-*c* and reduced that of Bcl-2 compared with controls. Tab neutralised these adverse effects of Dex.

### Effects of Tab on bone histopathology and BMD

**Fig. 9:**
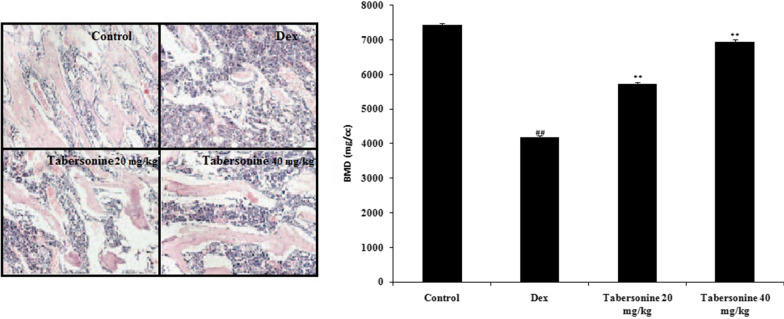
Tabersonine ameliorated Dex-induced osteoporosis in rats. **a** Histopathological bone changes revealed by H&E staining (100×). **b** BMD in rats with Dex-induced osteoporosis treated or not treated with Tab. Means ± SEMs (*n* = 10). ^##^*p* < 0.01 vs. control, **p < 0.01 vs. Dex group

Tab affected bone histopathology and BMD in rats with Dex-induced osteoporosis (Fig. 9). The control group exhibited normal bone. Dex destroyed bone trabeculae (as revealed by staining) and reduced bone synthesis. Tab neutralised these negative effects. The BMD was significantly lower in the Dex group than in the control group; Tab largely neutralised this deterioration.

## Discussion

Bone homeostasis is affected by osteoporosis, a disease characterised by the dysregulation of bone formation and resorption. Osteoblasts regulate bone formation and osteoclasts control bone resorption (Feng and McDonald 2011). Dex compromises osteoblast function; Dex-induced models of osteoporosis are used widely for the pharmacological evaluation of potentially useful anti-osteoporosis drugs (Yin et al. 2019). We employed a Dex-induced osteoporosis model to explore the effects of Tab on osteoblast viability, ALP activity, mitochondrial superoxide and ROS production, the MMP and apoptosis (the latter two parameters were assessed by flow cytometry). We performed RT-PCR and western blotting to explore the effects of Tab on osteoblast protein expression.

Mitochondria play important roles in cell survival and apoptosis (Wang and Youle 2009). Dex affects mitochondrial respiration by inducing ROS synthesis, in turn activating the cytochrome-*C*-associated pro-apoptotic pathway and the caspase cascade (Liu et al. 2018). Tab treatment enhanced the viability of Dex-treated osteoblasts and ALP activity, and reduced mitochondrial superoxide and ROS production. Tab attenuated the expression of caspase-3, Bcl-2, Bax, and cytochrome-*c* in Dex-treated osteoblasts. Flow cytometric analysis revealed that Tab enhanced the MMP and reduced apoptosis of Dex-treated osteoblasts.

Nrf2 signalling protects osteoblasts (Sena and Chandel 2012). The mitochondrial ROS level is regulated by Ho-1, NQO-1, and Nrf2 (Li et al. 2014). In models of osteoporosis, the Nrf2 pathway is suppressed, accompanied by mitochondrial dysfunction (Huang et al. 2019). We observed higher levels of Nrf2, Ho-1 and NQO-1 in the Tab groups than in the Dex group. The levels of mRNAs encoding RUNX2, BMP-2 and osterix were significantly enhanced in the Tab-treated groups compared with the Dex-treated group (all *p* < 0.01). In conclusion, our findings suggest that Tab ameliorates Dex-induced osteoporosis by regulating Nrf2/ROS/Bax signalling. Thus, Tab may be clinically valuable for osteoporosis management.

## Data Availability

The supporting data are not available for ethical reasons.
